# Adaptive cueing strategy for gait modification: A case study using auditory cues

**DOI:** 10.3389/fnbot.2023.1127033

**Published:** 2023-03-23

**Authors:** Tina L. Y. Wu, Anna Murphy, Chao Chen, Dana Kulić

**Affiliations:** ^1^Department of Electrical and Computer Systems Engineering, Monash University, Clayton, VIC, Australia; ^2^Clinical Research Centre for Movement Disorders and Gait, Monash Health, Cheltenham, VIC, Australia; ^3^Department of Mechanical and Aerospace Engineering, Monash University, Clayton, VIC, Australia

**Keywords:** continuum care, wearable robotics, rehabilitation robotics, human-in-the-loop, Parkinson's disease

## Abstract

People with Parkinson's (PwP) experience gait impairments that can be improved through cue training, where visual, auditory, or haptic cues are provided to guide the walker's cadence or step length. There are two types of cueing strategies: open and closed-loop. Closed-loop cueing may be more effective in addressing habituation and cue dependency, but has to date been rarely validated with PwP. In this study, we adapt a human-in-the-loop framework to conduct preliminary analysis with four PwP. The closed-loop framework learns an individualized model of the walker's responsiveness to cues and generates an optimized cue based on the model. In this feasibility study, we determine whether participants in early stages of Parkinson's can respond to the novel cueing framework, and compare the performance of the framework to two alternative cueing strategies (fixed/proportional approaches) in changing the participant's cadence to two target cadences (speed up/slow down). The preliminary results show that the selection of the target cadence has an impact on the participant's gait performance. With the appropriate target, the framework and the fixed approaches perform similarly in slowing the participants' cadence. However, the proposed framework demonstrates better efficiency, explainability, and robustness across participants. Participants also have the highest retention rate in the absence of cues with the proposed framework. Finally, there is no clear benefit of using the proportional approach.

## 1. Introduction

Parkinson's Disease (PD) is a progressive neurological disorder that causes a decline in motor capabilities. In advanced stages of PD, a key symptom known as Freezing of Gait (FoG) impairs people's ability to initiate, sustain, and control gait patterns, which reduces their quality of life (Sweeney et al., 2019). Cueing training can be helpful in improving gait performance, where people can use visual, auditory, or haptic cues to guide them on where/when to step (Ginis et al., 2018; Sweeney et al., 2019), thereby reducing the frequency of freezing and improving the temporal and spatial gait parameters such as speed, step length, and cadence (Nieuwboer et al., 2007).

Two types of cueing strategies have been identified: open or closed-loop (Muthukrishnan et al., 2019). The open loop strategy provides cues in a fixed manner that do not change regardless of the person's response. The fixed nature of the cue can be the constancy (e.g., visual cues at fixed distance, auditory/haptic cues at fixed pace) or its presence (i.e., always on). The effectiveness of open-loop strategy has been validated extensively in a variety of settings with PwP [e.g., home (Nieuwboer et al., 2007)/clinic (McCandless et al., 2016), short/long term (Lirani-Silva et al., 2019)], showing that open-loop strategies can be effective in improving gait parameters in PwP. However, key weaknesses of open-loop cueing include cue-dependency, where participants start to rely on cues, or habituation, where cues become less salient (and therefore less effective) as participants get used to the cues (Ginis et al., 2018).

Compared to open-loop, closed-loop strategies adjust cues based on the participant's real-time performance, which may address cue-dependency and habituation, and potentially provide greater gait and postural improvement (Mancini et al., 2018). Closed-loop cueing requires the participant's gait performance to be quantified, which can be measured using smartphones and/or wearable devices (e.g., Ginis et al., 2016; Chomiak et al., 2019). With the gait-monitoring capabilities, cues can be provided on-demand, only when symptoms of freezing occur (Ginis et al., 2016), or can be synchronized to each step (Mancini et al., 2018). However, many of these methods focus on the events leading up to the cue provision. There is a lack of adaptation to change the feedback based on the user's response. One example of cue adaptation is Zhang et al. (2020), where the speed of the cues is adjusted using a proportional-integral controller to minimize the difference between the user's walking speed and target speed. Our previous work proposed a human-in-the-loop (HIL) optimization strategy that models the participant's real-time response to cues (Wu et al., 2021). Recently, Zhang et al. (2022) developed a framework that can first estimate the user's maximum walking speed online, then uses reinforcement learning and fuzzy logic to adapt an intermediate, guiding speed to help the participant reach their maximum speed. While all the aforementioned adaptation strategies have been effective in changing healthy participants' gait performance, they have not yet been validated with PwP or other representative groups (Mancini et al., 2018).

We adapt the HIL framework and the study methodology originally evaluated with healthy participants in Wu et al. (2021) in this case study with PwP. In the HIL framework, a model of the person's response to cues is learned online using a Gaussian Process (GP). The GP model is then used in an optimization function to generate cues to improve gait performance. Compared to our previous work, this work evaluates adaptive cue generation for PwP and provides an analysis of the cue-selection mechanism. The study utilizes auditory cues due to the low development complexity (i.e., only needing a speaker, compared to visual/haptics cues that require other hardware). However, the framework generalizes to other cueing modalities. As a feasibility study, it is also important to determine whether PwP in the early stages of the disease can respond to cues.

Compared to the work by Zhang et al. (2022), the HIL framework can provide insights into the person's response to cues by explicitly modeling the response using GPs. The optimization framework guarantees that the selected cue is optimal and personalized given the person-specific cue response model. Finally, the HIL framework may allow for more practical clinical use, as the therapist would only need to select a target cadence, rather than defining the fuzzy rules that are needed for Zhang et al. (2022). The target selection being grounded in clinical metrics and the ability for person-specific adaptation are also the advantage of the HIL framework over the PI controller approach in Zhang et al. (2020), as the controller gains do not have meaningful clinical associations and lack the ability to adapt to the user's real-time condition.

## 2. Materials and methods

### 2.1. Summary of proposed framework

**Figure 1:**
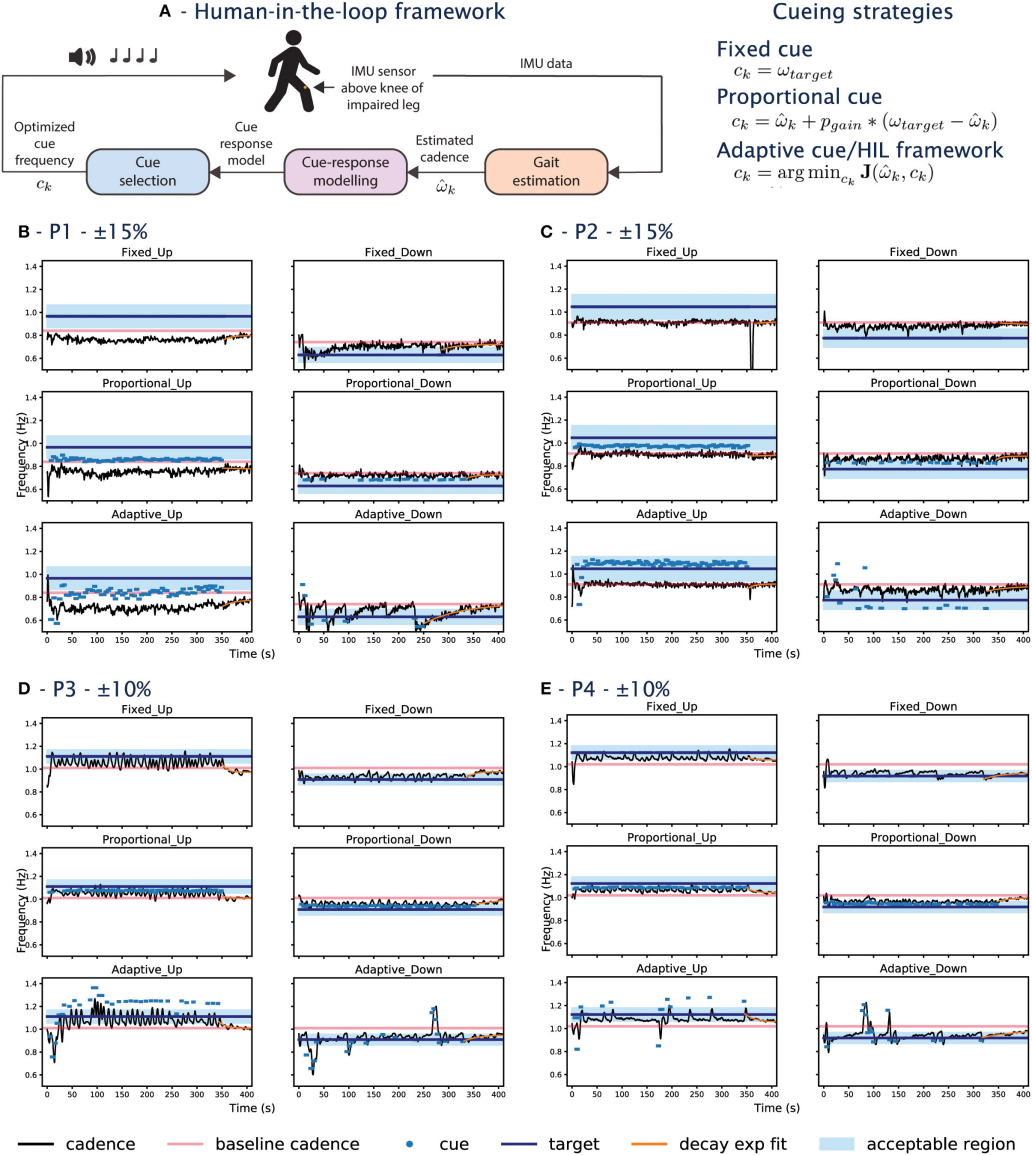
**(A)** HIL framework block diagram and the cueing strategies tested in the experiment. **(B–D)** Shows data from participants during the experiment. The x-axis shows the time in seconds and the y-axis is the frequency. **(B)** Corresponds to P1, **(C)** for P2, **(D)** for P3, **(E)** for P4. For each participant, the left column shows the UP conditions, and the right column are the DOWN conditions. The first row shows the fixed conditions, the second row the proportional conditions, and the third row are the adaptive conditions. The goal of the cueing approach is to bring the person's cadence (black line) into the blue-shaded acceptable region. The pink line shows the participant's baseline cadence. The dots show the frequency of the cues when they are played. The target cadence is listed at the top for each participant.

The HIL framework consists of three subcomponents: estimating gait parameters online, learning the individualized cue-response model, and providing cues using an optimization function. The framework block diagram is presented in Figure 1A. An Inertial Measurement Unit (IMU) sensor is used to capture cadence as the main gait performance metric. Cadence in Hertz (Hz) is estimated using the canonical dynamical system (CDS) proposed by Petrič et al. (2011).

To learn the individualized cue-response model, a Gaussian Process (GP) is used to learn the relationship between the current cadence as a function of the past cadence and past cue frequency as shown in Equation (1).


(1) $$\begin{array}{l} X=\left[\begin{array}{c} {\hat{\omega }}_{k-1},c_{k-1} \end{array}\right],\text{for }k\in (0,1,...,K-1)\;\\ Y=\left[\begin{array}{c} {\hat{\omega }}_{j} \end{array}\right],\text{for }j\in (1,2,...,K)\;\\ Y=f(X)+\beta ,\;\\ \text{where }f(X)\sim GP(m(X),cov(X,X^{\prime })) \end{array}$$

**X** and **Y** are the input-output data pairs of the GP, with a total of *K* number of pairs. ${\hat{\omega }}_{k}$ is the CDS estimate at the current sampling index *k*, which happens every four strides roughly at heel strike. **Y** consists of (${\hat{\omega }}_{j},j\in \left[1,2,...,K\right]$) is a vector of current cadence as the GP output data up to the current index *k*. **X** consists of a vector of past cadences (${\hat{\omega }}_{k-1}$) and past cues (*c*_*k* − 1_) up to index *k* − 1, which are the GP input data. The GP has a mean function, *m*(**X**), a covariance, *cov*(**X**, **X'**), and a constant basis β and is trained online and used to predict how the participant would respond to a given cue using Equation (2). This approach is inspired by previous works implementing the HIL framework for exoskeletons (e.g., Kim et al., 2017; Zhang et al., 2017).

Finally, a cost function is minimized to provide feedback as shown in Equation (3) and subject to the constraints in Equation (4).


(2) $$\begin{array}{l} {\hat{\bar{\omega }}}_{k+1}\left({\hat{\omega }}_{k},c_{k}\right)=f\left({\hat{\omega }}_{k},c_{k}\right)+\beta \end{array}$$

(3) $$\begin{array}{l} J({\hat{\omega }}_{k},c_{k})={\left(\omega_{target}-{\hat{\bar{\omega }}}_{k+1}({\hat{\omega }}_{k},c_{k})\right)}^{2}\;\\ \;c_{k}=\underset{c_{k}}{\text{arg min}}\;J({\hat{\omega }}_{k},c_{k}),\text{subject to} \end{array}$$

(4) $$\begin{array}{l} \max\left(-20\%\omega_{k},-35\%\omega_{baseline}\right)\leq c_{k}\leq \min\left(+20\%\omega_{k},+35\%\omega_{baseline}\right) \end{array}$$

Where *J* is the cost function that minimizes the squared difference between the target cadence and the predicted cadence to provide feedback, *c*_*k*_. The cost function is subject to the constraints in Eq 4, with bounds of ±20% of the current cadence and the ceiling/floor at ±35% of the person's baseline cadence (ω_*baseline*_). The constraints for the cost function were reduced compared to our work in Wu et al. (2021). The constraint prevents the cues from changing to the theoretical maximum/minimum in one iteration to prevent participants from needing to change their gait rapidly which may increase tripping risks. At runtime, the optimizer relies on the GP having learned the cue-response model, which requires training samples. A random exploration is initiated at the start of the experiment for each participant to collect samples until the gradient can be estimated, which leads to two-phase behavior that we called the exploration and the converged phases. The GP is trained continuously regardless of the phase, but we analyze the performance of the framework with respect to these two phases, where the exploration is the first 70 seconds of the experiment, and the converged phase is as the GP prediction error becomes sufficiently low as shown in **Figure 3J**.

### 2.2. Experimental conditions

#### 2.2.1. Cueing strategies

The experiment compares three different cueing strategies: fixed, proportional, and adaptive. The fixed cue condition provides cues with frequency at the target cadence, representing the current state-of-the-art open-loop cueing strategy. In the proportional condition, cue frequency proportional to the error between the current cadence and the target cadence is provided. The proportional condition represents a one-size-fits-all closed-loop cueing strategy as a constant error gain is used for all participants. The error gain was set to 0.5, as determined empirically in Wu et al. (2021). Finally, the adaptive cue provides cues using the HIL framework described in Section 2.1, which allows personalized cues to be provided. A summary of the cueing strategies can be found in Figure 1A.

#### 2.2.2. Target cadence selection

As the PD participants of the current study do not experience gait impairment, the goal is to alter their baseline walking to a new cadence. This is similar to the usage of cues during FoG, where cues are used to guide the patient to a non-freezing pace. Two target cadences (i.e., UP/DOWN speed conditions) are set based on each participant's baseline cadence, ω_*baseline*_. In this study, the targets are ±15*%ω*_*baseline*_ for the first two participants. We observe during the experiment that both participants were unable to respond to the +15% conditions and lower the targets to ±10*%ω*_*baseline*_ for the next two participants. The targets have been used in previous studies (e.g., Arias and Cudeiro, 2008; Hoppe et al., 2020). To account for natural variation in the walking and the gait estimation error, the target cadence constraint is relaxed to an acceptable boundary during implementation. This means cues would only play if the participant's cadence falls out of the boundary. The bounds are ±10*%ω*_*target*_ for the first two participants with ±15*%ω*_*baseline*_ as the target. The next two participants have the ±10*%ω*_*baseline*_ as the targets with the bounds of ±5*%ω*_*target*_. The acceptable boundary tightens for the later two participants to avoid the overlap with the baseline cadence. The change in acceptable boundary range enforces a minimum of 5% cadence change for all participants. The acceptable boundary check occurs every 4 strides, during which 8 beats are provided, to allow time for the participant to converge to the new cadence. The combination of targets and cueing approaches result in a total of 6 experimental conditions.

### 2.3. Participants

Four participants with PD were recruited by a clinician at the Movement Disorder Clinic, Kingston Center. Participants needed to have Hoehn and Yahr score^1^ of less than or equal to two regardless of medication state to participate and have no hearing impairments/allergies to adhesives. The criterion excludes those who experience gait impairment based on the clinician's assessment (i.e., freezing of gait, tremor in the lower legs, may be at risk of falls) in this feasibility study. All participants were tested during their subjective medication-ON state (if they are on medication). The study (ID 22556) was approved by the Monash University Human Research Ethics Committee.

### 2.4. Protocol

Participants watched an introductory video at the start of the experiment. Participants provided consent once they had a chance to have their questions addressed. Different from the previous study, the IMU sensor was fixed onto what participants self-reported as the more disease-affected leg. A familiarization session was provided where participants practiced syncing to 88 beats per minute. Instructions were given to sync each step to a beat. Afterwards, participants were told walk at their comfortable and natural pace for 7 minutes to measure ω_*baseline*_. Participants filled in a demographic survey after the baseline measurement. The 6 experiment conditions were then provided in random order and blinded from the participants. During the experiment, cues were played based on the condition described in Section 2.2 for the first 6 min (as per the standard 6-min Walk Test). No cues were played in the last minute of the condition to examine retention. Participants were instructed to sync their walking to cues to the best of their ability, but were not explicitly told to maintain the new cadence in the absence of beats. The researcher walked a few steps behind the participant throughout the experiment to provide support if needed. Participants were given breaks while they filled in a survey between experiments, which included filling in a NASA Task Load Index (TLX) and if/how the participants thought their gait changed during each experiment. The break was extended on demand to avoid fatigue. Once participants completed all experiment conditions, an exit interview was conducted to fill in a system usability scale (SUS) and gather information on the participant's qualitative experience, followed by a debrief session. The debrief explained the conditions of the experiment and the participants also had a chance to review their data in relation to the goal and the experimental conditions. The study took 1.5 hours to complete.

### 2.5. Materials

The same setup in Wu et al. (2021) was used and summarized below. The motion data was recorded using an IMU sensor from the WaveTrack Inertial System at 285 Hz (Cometa Systems, Milan, IT) and streamed wirelessly into a custom C# program. The C# program ran on a laptop (Windows 10, i7 core with no GPU), which controlled the timing of the auditory cues played from a speaker (Phillips BT50A). The program also interfaced with MATLAB, where the Statistics and Machine Learning and Optimization Toolboxes were used for the HIL framework. The GP was initialized with X = [0,0], Y = [0] for each participant.

### 2.6. Analysis

No statistical analysis was conducted due to the small sample size. The analysis will focus on reporting the individual raw data and metrics, combined with the participant's subjective ratings.

## 3. Results

### 3.1. Demographics

Four participants enrolled in the study (2M/2F; age 70 ± 5; height 165.25 ± 12.1; weight 64.25 ± 16.31; years since diagnosis 3.5 ± 1.5). The participants' self-reported lower body symptoms are as follows, with the number of participants who reported in brackets: stiffness (1), slowed movement (2), trouble balancing (2), and dyskinesia (1). One participant does not experience any symptoms and others indicated that these symptoms rarely occur. All participants exercise 3+ times a week, with the most common exercise being walking (4), strength/resistance training (2), and stretching/balance training (2). No participant had prior experience with strategy training (i.e., visual cueing, audio cueing, haptic feedback, vibration therapy).

### 3.2. Cueing strategies comparison

#### 3.2.1. Mean percent change from baseline

**Figure 2:**
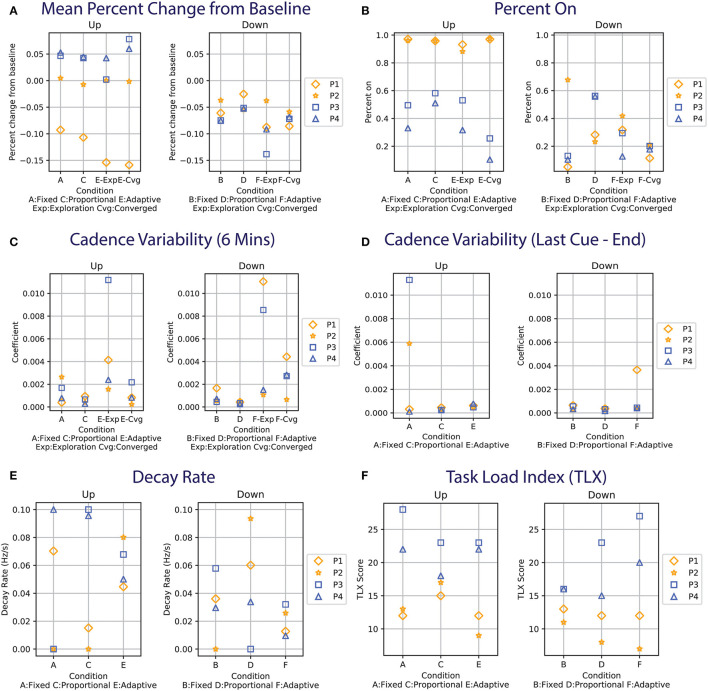
**(A)** Mean percent change from baseline for each participant. For P1&P2 (shown in yellow), ω_*target*_ is 15%. ω_*target*_ is 10% for P3&P4 (in blue). The left panel shows the UP conditions and the right are the DOWN conditions. Data with similar values may be overlapping (e.g., P2&P3 in E-Exp, Up). **(B)** Percent on time during the first 6 min of the experiment. **(C)** Cadence variability computed using the coefficient of variation during the first 6 min of the experiment. **(D)** Cadence variability during the retention phase (last minute of the experiment). **(E)** Decay rate computed by fitting an exponential function to the data from the last provided cue (see orange lines in Figures 1B–D). **(F)** Task Load Index for the participants.

The mean percent change from the baseline (${\bar{\delta }}_{BL}$) is shown in Figure 2A. This metric is an indicator of whether the approach is able to sustainably influence the person's cadence. We hypothesize that a positive percent change from baseline will be observed in the UP conditions and a negative percent change for the DOWN conditions. For instance, the ${\bar{\delta }}_{BL}$ for P1&P2 (in yellow) would be close to +0.15 in the right panel and –0.15 in the left panel in Figure 2A. The comparison focuses on the converged adaptive cue after the model has been learned in the exploration phase.

In the UP conditions, P1's cadence does not reach +0.15 regardless of the cueing approach. The behavior can also be seen in the raw data, where the participant's cadence (black line) is always below the baseline cadence (pink line) for the UP conditions (left column) in Figure 2B. For the DOWN conditions, while the target of –0.15 was not reached for all approaches, the adaptive-DOWN condition showed the largest mean percent change from the target as seen in Figure 2A.

The cadence for P2 is similar to the baseline across all UP conditions (Figure 2A), even during the exploration phase of the adaptive condition where a series of random cues is provided. This is illustrated in Figure 1C, where the random cues are seen within the first 50 s of the adaptive approach (last row), but the participant's cadence tracks the baseline (i.e., the black line oscillates around the pink line). The largest change is seen around 350 s in the Fixed-UP condition in Figure 1C. The cadence dropped because it was the first condition and the participant paused walking when the cue stopped playing for the first time. The most significant change in ${\bar{\delta }}_{BL}$ is in the adaptive-down condition, where P2 responded best to the slower cue provided in the adaptive condition compared to the fixed/proportional approach.

For P3&P4, the target is set to ±10%, meaning the blue shapes in Figure 2A should ideally be close to +0.1/–0.1 for the right/left panel. The target was lowered for P3&P4 after observing that the first two participants were unable to reach the fast target. The adaptive approach for P3&P4 in both UP/DOWN conditions is comparable to the state-of-the-art fixed approach in terms of ${\bar{\delta }}_{BL}$. In the UP condition, the converged adaptive approach achieves the highest mean percent change from baseline for P3 and P4, especially for P3 as the participant had the highest ${\bar{\delta }}_{BL}$ among all conditions and all participants.

#### 3.2.2. Percent on

The percent on metric examines the percentage of time the cue is provided in the first 6 min of the study, which is shown in Figure 2B. The metric is a measure of cue efficiency. The participant's cadence did not converge to the cues despite the constant presence of cues in the UP conditions for P1&2. For P3&4, the adaptive-UP condition requires the least percent on time to keep participants at the target boundary.

In the DOWN conditions, the converged adaptive condition (F-Cvg in Figure 2B) shows good performance across all participants. Despite the fixed-DOWN (Figure 2B) condition having the lowest percent on time among the three, it is ineffective for P2. The proportional cue has a similar percent on time between the UP/DOWN conditions for P3&4, but is drastically different between the UP/DOWN conditions for P1&2 (i.e., always on for UP, relatively low for DOWN).

#### 3.2.3. Gait variability

The coefficient of variation of the participant's cadence is computed for the first 6 min of the experiment (Figure 2C) and during the retention phase from the last cue to the end (Figure 2D), following the definition in Lo et al. (2017). The variability is the highest during the adaptive condition exploration phase when cues with the largest variance are provided (see Figures 1B–E). The proportional approach has the lowest variability, as cues of similar frequencies are provided. In the fixed/converged-adaptive approaches, the variability is similar in the UP conditions, but the adaptive DOWN has a higher variability. This might be due to the adaptive approach undergoing a second exploration, which happened in both P3&4. P1's variability s also high in the adaptive down condition as the participant's cadence experience sharp changes (sudden dip in cadence when cues are provided, see Figure 1B).

During the retention phase when no cue was played, the cadence variability is generally low except for P3 in Fixed UP, P2 in Fixed UP, and P1 in Fixed DOWN. P3 had the highest variability as the participant's cadence varied from the UP target to a value lower than the baseline. P2 had a high variability in Fixed UP due to the participant stopping/starting as described in Section 3.2.1. While a manual offset is applied to skip the pause/start, the data should be considered an outlier. P1 in Adaptive DOWN also had high variability due to the larger variance around t=350s. Overall, the variability is higher when cues are played, as participants actively change their cadence to match the cue. The variability is immediately decreased when there is no cue.

#### 3.2.4. Decay rate

An exponential function of the form of *y* = α*e*^ − η*x*^+γ is fitted to the cadence data from the last provided cue to the end of the experiment to examine the rate at which participants settle to a cadence in the absence of cue. The absolute value of the decay rate, η, is presented in Figure 2E. The function is plotted in orange in Figures 1B–E. Participants can settle back to their baseline cadence (e.g., P3, Proportional UP in Figure 1D), a faster cadence (e.g., P4, Adaptive UP in Figure 1E), or a slower cadence (e.g., P1, Adaptive UP in Figure 1B). A higher value would indicate a faster settlement. A low value indicates a minimal change from the baseline or from a new cadence.

Overall, the decay rates for the fixed and proportional approaches in UP have a higher variance compared to the adaptive condition. The results can be attributed to participants not being responsive to cues (e.g., P1&P2) or the fast pace being hard for participants to maintain (as the cadence for P3&4 both drop significantly once the cue is removed). There is no clear difference between the cueing approaches in the UP conditions. In the down condition, the adaptive approach on average has the lowest decay rate, as well as the lowest variance. Since participants are all able to slow down, the result suggests that participants are able to better maintain the new DOWN target using the adaptive approach. The proportional approach in the DOWN condition again had the largest variance and the fastest average decay rate.

### 3.3. Adaptive framework cue-selection mechanism: Strengths and limitations

**Figure 3:**
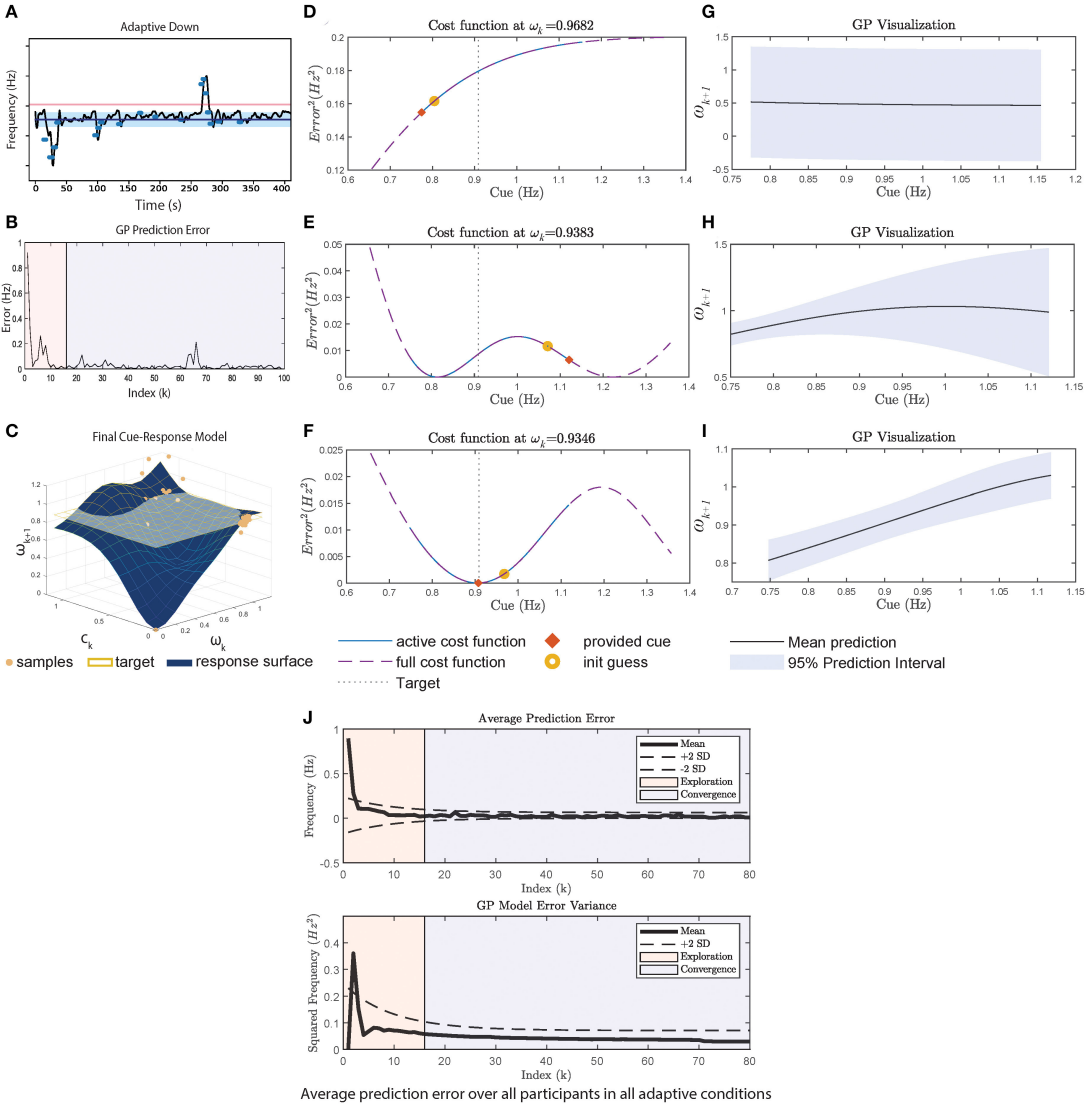
A case study using data from P3 adaptive-DOWN condition in Figure 1C. **(A)** Cadence over time in the experiment. **(B)** The GP prediction error, which is calculated at every 4 strides. **(C)** Shows the final 3D cue response model, where the X axis is ω_*k*_, Y axis is *c*_*k*_ and the Z axis is ω_*k*+1_. Samples are plotted in blue circles. ω_*target*_ is plotted as the yellow horizontal plane. **(D–F)** Shows the cost function at the start, middle, and end of the experiment. The middle part is selected to explain the second exploration phase. The cost function is plotted over the absolute maximum and minimum cue range (dotted purple line), where the active cost function defined by Equation (4) is plotted as solid blue. The initial guess provided to the optimizer is plotted as a yellow circle, and the final provided cue is an orange square. **(D)** Shows the cost function at the start. **(E)** Shows the selection of cues given the poor representation and poor optimizer's initial guess. **(G)** Shows the final shape of the cost function, which has a minimum that is close to ω_*target*_. **(G–I)** Shows the GP mean and variance at start/middle/end. **(J)** The average GP prediction error for all participants. The exploration phase (i.e., first 70 seconds) is shaded in pink. The prediction error and variance decrease significantly during exploration.

The mechanism of the adaptive framework is discussed in this section with illustrations shown in Figure 3, considering the Adaptive-DOWN condition for P3, shown in Figure 3A. The condition is interesting as the framework undergoes 2 exploration periods (once at the start and once at t≈260s).

The initial exploration phase lowers the prediction error as seen in Figure 3B, until reaching t≈260s, k≈60 in B. GP undergoes another exploration period until the prediction error settles again. In the analysis, we visualize the cost function at each ω_*k*_ at *k* = 5, 60, 94 (index for start/second exploration/end) (Figures 3D–F) and the corresponding GP realization (Figures 3G–I).

At the start, the GP posterior mean prediction starts off flat across the range of cues as shown in Figure 3G. During this phase, the constraints defined in Equation (4) are in effect, causing cues at the maximum/minimum to be provided, as shown in Figure 3D.

Between 0 and 250 s in Figure 3A, cues with frequency of <1 Hz are provided. Through repeated sampling, GP captures the participant's response and the prediction variance also decreases significantly between Figures 3G, H in the <1 Hz region. This prediction variance remains large for cues>1 Hz due to the lack of samples. This demonstrates a limitation of the random initial exploration technique as it does not adequately cover the state space, which results in a poor model in regions where there are insufficient samples. The large uncertainty and the optimizer's initial guess leads to the second exploration phase, where the upper range of the cue is explored, resulting in a cue at the upper bound to be provided.

The GP visualization toward the end of the session is plotted in Figure 3I. Once the GP model has low variance across the range of cues, the cost function minimum approaches ω_*target*_ = 0.9 as seen in Figure 3F, since the participant is generally responsive to cues (i.e., able to walk at the pace of the cue). However, the participant does not synchronize exactly to the cue, so a cue slightly below the target is more effective. Similarly for the UP condition, the adaptive framework provides cues at a much higher pace than the fixed approach. In general, the minimum of the cost function represents the participant's best performance in relation to the target given that the GP has been adequately explored. The continuous learning ability of the adaptive framework may provide a mechanism to handle habituation as the framework can alter the cues based on the current user model. In addition, the explicit modeling of the user's response to cues through GP may provide clinicians with insights about changes in the user's motor capability and users with better transparency and explainability. The final cue-response surface of P3 is plotted in Figure 3C.

### 3.4. Subjective rating and responses

The Task Load Index (TLX) is administered after each condition and the result is plotted in Figure 2F. Overall, P1&2 rated the mental workload for the experiment to be lower compared to P3&4. The UP and DOWN conditions have similar TLX scores (UP: 17.83 ± 1.4, DOWN: 15 ± 1.75). The adaptive condition has the lowest mean in the UP condition despite P3&4 both experiencing two phases of rapid cue changes. The result is not due to practice effect. As stated by a participant in the post-study interview, cue synchronization ‘'[becomes] like a game.” In each post-condition survey, participants are asked whether their walking changed compared to normal (in the form of yes/no). Out of the 24 total experimental conditions across the 4 participants, there are 9 reports (37.5%) for no change in walking (2 Fixed-UP, 1 Fixed-DOWN, 4 Proportional-UP/DOWN, 2 Adaptive-UP). All participants answered “no” in at least one condition. All participants felt their gait changed in adaptive-DOWN (i.e., answered “yes”). PD participants reported no change at a much higher rate compared to the previous study with healthy participants (19/150 = 12.7% reported no change; the target is ±20%). In the post-study interview, P1&2 both mentioned that they thought their natural cadence matched the UP targets, which was surprising given that these two participants failed to increase their cadence in response to any cue strategy. The overall cue-provision system scored an average of 78.75 ± 13.77 out of 100 on the system usability scale using the calculation detailed in Brooke (1996). Most participants found the system easy to use, would like to use the system frequently if they need it in the future but emphasize on the need for help during initial setup.

## 4. Discussion

In this experiment, we first evaluate whether participants can respond to the cue, especially when cues are not fixed. The results show that all three cueing approaches are able to influence the participants' cadences in the DOWN conditions. Specifically, the adaptive approach performs similarly to the benchmark fixed approach in slowing the cadence. The benefit of the adaptive approach is the efficiency and robustness across participants, meaning it does not cue the participants as frequently in the converged phase and works equally well across participants as seen by the low variance in the data. In addition, the adaptive approach in the DOWN cases has the best retention given by the lowest decay rate. The better retention could be attributed to the adaptive condition providing slower cues compared to the other two strategies, combined with the target being feasible to maintain. Clinically, the impaired ability to regulate step length could be the fundamental challenge in PD, which is compensated by the increase in cadence (Morris et al., 1994). In Morris et al. (1994), the authors observed improvement in stride length in the −10*%ω*_*target*_ speed condition. While later studies have also shown other metronome settings to be effective (e.g., Willems et al., 2006; Arias and Cudeiro, 2008 tested ranges from −70% to +120%), the implication of the current study is that by successfully modulating the participant's cadence, the step length may be improved. Another clinical aspect examined is the cadence variability. While cues generally increase variability (typically associated with the increased risk of falls Lo et al., 2017), the variability is an intended study effect as participants need to actively change their pace. In addition, the variability decreases immediately in the absence of cues, which demonstrates the short-lasting effect in a single-session study.

As mentioned by Zhang et al. (2022), the target speed selection during gait rehabilitation is still an open research question. In our experiment, the performance of the cueing approaches was impacted by the selection of the initial target, as seen especially in the UP +15*%ω*_*baseline*_ case for P1&2, where all three cueing strategies were unsuccessful in influencing the participants' cadences. When participants are not responsive to cues, the benefit of the adaptive approach is allowing a better understanding of the participant's response landscape. Overall, there is no clear benefit of the proportional approach based on the metrics of the study.

**Figure 4:**
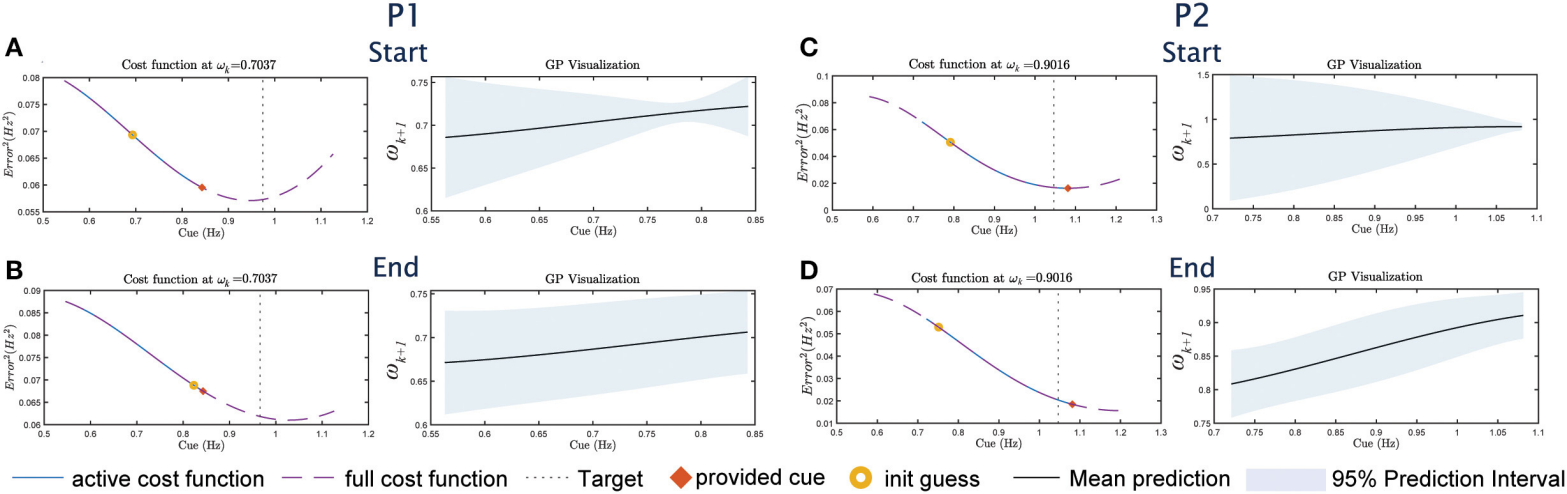
The cost function/GP visualization sets for P1&2 for UP, following the format in Figures 3D–J. For P1, **(A)** shows the visualization at the start of the study, **(B)** shows the visualization at the end of the study. For P2, **(C)** shows the visualization at the start and **(D)** at the end.

The GP model combined with the cost function results in the optimal selection of cue given the constraint in Equation (4). This is illustrated in Figure 4, where the provided cue (marked in red x) is the minimum within the constraint boundaries. However, the boundaries represent a trade-off between safety and convergence. By limiting the change in cue amplitude, participants do not need to rapidly change their cadence, which reduces the risk of tripping. However, the boundary prevents the global minimum from being selected (as illustrated by the dashed lines in Figure 4). Since P1&2 did not change their cadence during the experiment, the adaptive approach is therefore stuck within the boundary in Eq. 4. The convergence of the GP as indicated by the variance is also not as drastic as in Figure 3I for P3. Overall, in addition to the reliance on the GP model, the adaptive framework performance may also be influenced by the optimizer's initial guess (as seen in Figure 3E), and the boundary constraint.

The experiment task (i.e., walking and syncing to the beats) may have been challenging due to dual-task interference and impaired beat perception, particularly for P1&2. Parkinson's disease disrupts the basal ganglia (BG) function in the brain, which is involved in both motor and other cognitive functions. Particularly, BG is used to carry out automatic, learned movements such as walking (Wu et al., 2015). In therapy, cueing can be used to bring attention to the walking task and therefore bypass the automatic control for walking. However, when walking while listening to the beats, the attention may be split between walking and keeping track of the beats, which may result in decreased performance for both tasks (i.e., dual-task interference). In addition, Schwartze et al. (2011) has shown that BG damages can lead to poor beat perception. PD participants also experience difficulty in acquiring new motor skills (i.e., sync walking to beats) (Rochester et al., 2010; Wu et al., 2015) and therefore participants may need more time to execute the new skills (Ghai et al., 2018), which may explain why P1&2 were unable to match the UP targets compared to the DOWN targets.

Potential confounding variables include fatigue and learning effect. Multiple participants requested a longer break as the experiment progressed, potentially indicating fatigue. Despite these factors, no trend is found when ordering the main metrics of the study by the experimental order, meaning the participant's performance did not get better or worse over time. This finding is consistent with our previous study with healthy participants. The major limitations of this study are the small sample size and the lack of an age-matched control group, which we plan to address in the follow-up study. In addition, further research will need to be conducted to bridge the perception gap potentially with different cueing modalities.

## Data availability statement

The raw data supporting the conclusions of this article will be made available by the authors, without undue reservation.

## Ethics statement

The studies involving human participants were reviewed and approved by Monash University Human Research Ethics Committee (ID 22556). The patients/participants provided their written informed consent to participate in this study.

## Author contributions

TW is responsible for conducting the studies, performing analysis, and writing the manuscript. AM is responsible for recruiting participants and providing clinical insights. CC is responsible for providing feedback on technical development. DK is responsible for overseeing the project and providing feedback on all components of the study and the manuscript. All authors contributed to the ideation of the study. All authors contributed to the article and approved the submitted version.

## Footnotes

^1^Hoehn and Yahr score (HY) is a clinical scale that is used to classify the motor function of Parkinson's Disease. The scale rating goes from 1 to 5 and the progression in the HY score corresponds to more severe motor impairment (Bhidayasiri and Tarsy, 2012). Functionally, HY ≤2 means participants can perform their daily routine independently or with minimal assistance.
